# Repetitive Peripheral Magnetic Stimulation of Wrist Extensors Enhances Cortical Excitability and Motor Performance in Healthy Individuals

**DOI:** 10.3389/fnins.2021.632716

**Published:** 2021-02-18

**Authors:** Mitsuhiro Nito, Natsuki Katagiri, Kaito Yoshida, Tadaki Koseki, Daisuke Kudo, Shigehiro Nanba, Shigeo Tanabe, Tomofumi Yamaguchi

**Affiliations:** ^1^Department of Anatomy and Structural Science, Yamagata University School of Medicine, Yamagata, Japan; ^2^Graduate School of Health Sciences, Yamagata Prefectural University of Health Sciences, Yamagata, Japan; ^3^Faculty of Rehabilitation, School of Health Sciences, Fujita Health University, Toyoake-shi, Japan; ^4^Department of Physical Therapy, Faculty of Health Science, Juntendo University, Bunkyo-ku, Japan

**Keywords:** corticospinal tract, intracortical circuits, plasticity, rehabilitation, spinal networks, upper extremity

## Abstract

Repetitive peripheral magnetic stimulation (rPMS) may improve motor function following central nervous system lesions, but the optimal parameters of rPMS to induce neural plasticity and mechanisms underlying its action remain unclear. We examined the effects of rPMS over wrist extensor muscles on neural plasticity and motor performance in 26 healthy volunteers. In separate experiments, the effects of rPMS on motor evoked potentials (MEPs), short-interval intracortical inhibition (SICI), intracortical facilitation (ICF), direct motor response (M-wave), Hoffmann-reflex, and ballistic wrist extension movements were assessed before and after rPMS. First, to examine the effects of stimulus frequency, rPMS was applied at 50, 25, and 10 Hz by setting a fixed total number of stimuli. A significant increase in MEPs of wrist extensors was observed following 50 and 25 Hz rPMS, but not 10 Hz rPMS. Next, we examined the time required to induce plasticity by increasing the number of stimuli, and found that at least 15 min of 50 and 25 Hz rPMS was required. Based on these parameters, lasting effects were evaluated following 15 min of 50 or 25 Hz rPMS. A significant increase in MEP was observed up to 60 min following 50 and 25 Hz rPMS; similarly, an attenuation of SICI and enhancement of ICF were also observed. The maximal M-wave and Hoffmann-reflex did not change, suggesting that the increase in MEP was due to plastic changes at the motor cortex. This was accompanied by increasing force and electromyograms during wrist ballistic extension movements following 50 and 25 Hz rPMS. These findings suggest that 15 min of rPMS with 25 Hz or more induces an increase in cortical excitability of the relevant area rather than altering the excitability of spinal circuits, and has the potential to improve motor output.

## Introduction

Peripheral nerve electrical stimulation is known to augment synaptic plasticity in motor cortex and spinal circuits in healthy individuals and in patients following stroke (Ridding et al., 2000; Kaelin-Lang et al., 2002; Khaslavskaia et al., 2002; McKay et al., 2002; Knash et al., 2003; Everaert et al., 2010; Mang et al., 2010; Chipchase et al., 2011a, b; Schabrun et al., 2012; Yamaguchi et al., 2012, 2013; Gallasch et al., 2015; Sasaki et al., 2017; Takahashi et al., 2018). Since synaptic plasticity is observed following rehabilitation and motor skill training, these changes may play an important role in the recovery (Nudo et al., 1996) and improvement of motor performance (Lotze et al., 2003; Perez et al., 2004; Tatemoto et al., 2019).

Repetitive peripheral magnetic stimulation (rPMS), as well as peripheral nerve electrical stimulation, can induce muscle contraction. However, magnetic stimulation is less painful than electrical stimulation, since the eddy current induced by magnetic stimulation directly stimulates deep tissues without penetrating the skin (Polson et al., 1982). Therefore, rPMS is less likely to induce discomfort, which is useful for patients in clinical settings. Previous studies have shown that rPMS improves motor dysfunction following central nervous system (CNS) lesions (Struppler et al., 2003; Flamand et al., 2012; Beaulieu and Schneider, 2013; Flamand and Schneider, 2014); however, stimulus parameters such as frequency and intervention time in these reports are not constant, and plastic changes in cortical excitability have not been directly investigated.

Gallasch et al. (2015) investigated the effects of two different frequencies of rPMS on cortical excitability, demonstrating that the cortical excitability of wrist flexor muscles increased following 25 Hz rPMS for 20 min, but not for 10 Hz rPMS. To align the intervention time, however, the number of stimuli was different for each frequency condition; therefore, it is unclear whether the cortical excitability changes induced by rPMS were frequency-dependent (Pitcher et al., 2003; Mang et al., 2010; Gallasch et al., 2015), dose-dependent (McKay et al., 2002; Andrews et al., 2013) or both. In addition, they reported that a facilitatory effect was observed following a series of rPMS for 20 min, but no study has examined the intervention timeframe of rPMS required to induce the changes in cortical excitability. If changes in cortical excitability are induced following rPMS, the effects may improve motor performance; however, these questions still remain unclear. This investigation has clinical implications for the application of rPMS in the rehabilitation of individuals with CNS lesions. The aim of this study was to investigate the effects of different rPMS parameters in wrist muscles on the excitability of cortical and spinal networks, and motor performance, in healthy individuals.

## Materials and Methods

### Participants

A total of 26 healthy volunteers participated in this study, 15 (seven females) with a mean age of 23.8 years (standard deviation: SD, 5.1 years) in Experiment 1, 14 (six females) with a mean age of 24.1 years (SD 5.1 years) in Experiment 2, 20 (nine females) with a mean age of 22.7 years (SD 5.1 years) in Experiment 3, and 20 (nine females) with a mean age of 23.4 years (SD 5.4 years) in Experiment 4. None of the participants had a history of neurological disease or were receiving any medication affecting the CNS. Sample size was determined on the basis of previous study investigating the effect of rPMS on cortical excitability (Gallasch et al., 2015). Each participant’s dominant hand was established using Chapman’s dominant hand test (Chapman and Chapman, 1987). All participants except two were right-handed. Experimental procedures were approved by the Ethics Committee of Yamagata Prefectural University Health of Sciences (approval number: 1806-06) and were performed in accordance with the Declaration of Helsinki. Prior to participation in this study, all participants signed written informed consent to the experimental procedures.

### Electromyogram Recording

The electromyogram (EMG) signals were recorded with a pair of Ag/AgCl disc surface electrodes (1.0 cm diameter). The electrodes (1.5 cm interelectrode distance) were secured to the skin overlying the right extensor carpi radialis (ECR) and flexor carpi radialis (FCR) muscles. The EMG signals were amplified, bandpass filtered (10–1,000 Hz), and sampled at 10 kHz using Neuropack (MEB-2306; Nihon Koden, Tokyo, Japan). The recorded signal was digitized for later analysis using LabVIEW software (National Instruments, Austin, TX, United States) in Experiments 1–3, and LabChart software (AD Instruments, Colorado Springs, CO, United States) in Experiment 4.

### rPMS

Repetitive peripheral magnetic stimulation was applied to the ECR muscle, as it has been reported that motor dysfunction is often observed after corticospinal tract lesions, such as following stroke (Mazevet et al., 2003; Koganemaru et al., 2010; Choudhury et al., 2019). The forearm was fixed in a pronation position with the fingers free. A biphasic pulse of rPMS was delivered using a figure-eight coil (Cool-B65; outer diameter 75 mm) connected to a MagPro R30 (MagVenture A/S, Denmark). The coil was placed on the skin overlaying the right ECR muscle with the handle pointing distal (i.e., toward the hand) and perpendicular to the forearm. The contraction of the ECR muscle was confirmed by palpation with the stimulus intensity well above the motor threshold (MT) of the direct motor response (M-wave). The MT was defined as the minimal stimulus intensity required to induce an M-wave by a single-pulse stimulus in at least three of five consecutive trials, and then the intensity was set at 120% of the MT. This intensity was expected to have a facilitatory effect on the cortical excitability, and to reduce the effect of fatigue (McKay et al., 2002; Chipchase et al., 2011a, b; Sasaki et al., 2017).

### Motor Evoked Potential

Transcranial magnetic stimulation (TMS) was applied over the left primary motor cortex using a figure-eight coil (loop diameter 70 mm) connected to two Magstim 200^2^ with a BiStim module (Magstim Company, Whitland, Dyfed, United Kingdom). We determined the optimal positioning to elicit a motor evoked potential (MEP) in the ECR muscle at rest (hot spot) by moving the coil with the handle pointing backward and 45° away from the midline. The hot spot was defined as the region where the largest MEP in the ECR muscle could be evoked with the minimum stimulus intensity (Lotze et al., 2003). The resting MT (rMT) was defined as the minimal stimulus intensity required to induce MEPs of 50 μV (peak-to-peak amplitude) in at least three of five consecutive trials in the relaxed muscle (Lotze et al., 2003). The intensity of single-pulse TMS was set at 120% of the rMT to measure MEPs as an indicator of corticospinal excitability. A total of 15 MEPs was recorded in the resting condition. These 15 measurements of the peak-to-peak MEP amplitude were averaged, and the mean value among participants was calculated. The average MEP value at each tested time point following rPMS was expressed as a percentage of the initial baseline measurement (normalized MEP) and used for statistical analysis.

### Short-Interval Intracortical Inhibition and Intracortical Facilitation

To induce short-interval intracortical inhibition (SICI) and intracortical facilitation (ICF), we used a sub-threshold conditioning paired-pulse paradigm (Kujirai et al., 1993). Two magnetic stimuli were supplied to the left primary motor cortex using the same stimulating coil. Conditioning stimulus intensity was set at 80% of the active MT (aMT) of the MEP response in ECR muscle. The aMT was defined as the minimal stimulus intensity required to induce MEPs of 200 μV in at least three of five consecutive trials while the participant performed isometric wrist extensions with an EMG amplitude of 100 μV. The test stimulus intensity was set at 120% of the rMT. Throughout the experiment, the test stimulus was adjusted to maintain an MEP amplitude equal to the ECR MEP amplitude at baseline. The inter-stimulus interval was set at 2.5 (SICI) and 10 ms (ICF) (Kujirai et al., 1993; Fisher et al., 2002), and 15 frames each were recorded of the paired-pulse and single stimulation conditions for each trial. The inter-stimulus interval of single TMS pulses was pseudorandom, between 4 and 6 s. The conditioned MEP amplitudes were expressed as percentages of the mean test MEP amplitudes. SICI values lower than 100% indicated inhibition, while ICF values greater than 100% indicated facilitation.

### M-Wave and H-Reflex

To induce M-wave and Hoffmann-reflex (H-reflex) of wrist extensors, electrical rectangular pulses of 1.0 ms duration were percutaneously delivered to the radial nerve trunk using bipolar surface electrodes (1.0 cm diameter, 1.5 cm interelectrode distance) placed on the nerve trajectory at the lateral intermuscular septum of the arm. The stimulus electrodes were connected to a Neuropack (MEB-2306; Nihon Koden). The inter-stimulus interval was 0.3 Hz.

To confirm fatigue as a result of rPMS (Sacco et al., 1997; Crone et al., 1999), the maximum M-wave (Mmax) was measured by supramaximal electrical stimulation (at an intensity of 120% to induce Mmax). The H-reflex recruitment curves were measured to assess the spinal network excitability. The number of participants that demonstrate recordable H-reflex of ECR are limited (Pierrot-Deseilligny and Burke, 2012; Burke, 2016); we were able to record the H-reflex at rest in 6 out of 15 participants in Experiment 1. The stimulus intensity was progressively increased by 0.2–1.0 mA from the minimum intensity necessary to elicit an H-reflex to the maximum amplitude of H-reflex (Hmax), and 7–10 responses were recorded at each stimulus intensity. Peak-to-peak amplitude of M- and H-waves were measured, and the mean value among participants was calculated.

### Experimental Procedure

Four experiments were conducted in order to investigate the following: (1) the effects of rPMS frequency on the excitability of corticospinal and spinal networks; (2) the effects of the dose or number of rPMS stimuli on corticospinal excitability; (3) the lasting effects of rPMS on corticospinal and cortical excitability (i.e., MEPs, SICI, and ICF); and (4) the effects of rPMS on motor performance. The procedures for each experiment are described in detail below. Throughout each experiment, the participants were comfortably seated, and the examined right arm lay on an armrest with the shoulder slightly flexed (approximately 10°), the elbow flexed (approximately 90°), and the forearm pronated.

#### Experiment 1: Effects of the Frequency of rPMS on MEPs, M-Waves, and H-Reflexes

**FIGURE 1:**
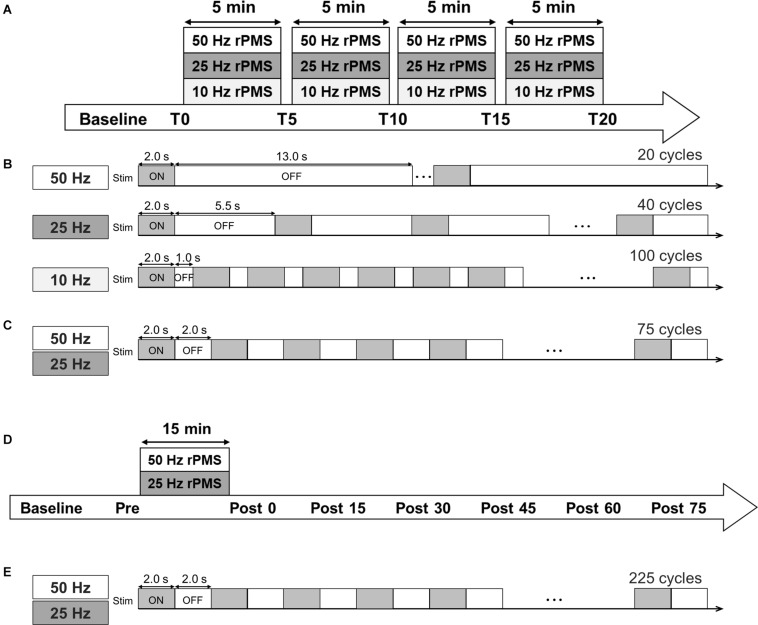
Experimental protocol. **(A)** Time course of Experiments 1 and 2. **(B)** Stimulation conditions for Experiments 1 and **(C)** 2. **(D)** Time course of Experiments 3 and 4. **(E)** In Experiments 3 and 4, three sessions (a total of 225 ON-OFF cycles) were performed.

The following were applied in random order of three separate sessions on different days: 50 Hz rPMS, 25 Hz rPMS, and 10 Hz rPMS. The sessions were separated by at least 1 day to prevent carry-over effects from the previous interventions. The total number of pulses was fixed based on the number of stimuli in the 10 Hz rPMS session (8,000 stimuli). In a single cycle, the stimulation was delivered for 2 s in all three frequency conditions, and the inter-stimulus interval and number of cycles was set so as to align the intervention time (Figure 1). The intervention consisted of four sessions (5 min per session), for a total of 20 min (Gallasch et al., 2015).

Motor evoked potentials were measured 5 min before intervention (baseline), just before rPMS (T0) and after one session (T5), two sessions (T10), three sessions (T15), and four sessions of rPMS (T20). To assess changes in spinal network excitability, Mmax and Hmax were measured at T0 and T20.

#### Experiment 2: Effects of the Stimulus Number of rPMS on MEPs

We applied 50 and 25 Hz rPMS to the ECR muscle on different days (at least 1 day apart) in a random order. In both conditions, the stimulation was delivered in a 2 s ON and 2 s OFF cycle, and the total number of cycles per single session was 75. A total of four sessions were performed (5 min per session, a total of 20 min) as in Experiment 1. The total number of pulses was 30,000 stimuli for the 50 Hz rPMS and 15,000 stimuli for the 25 Hz rPMS. MEPs and Mmax was measured as in Experiment 1.

#### Experiment 3: Lasting Effects of the rPMS on MEPs, SICI, and ICF

We applied 50 and 25 Hz rPMS to the ECR muscle on different days (at least 1 day apart) in a random order. The stimulation was delivered in a 2 s ON and 2 s OFF cycle for 15 min, consisting of 225 cycles. MEPs were measured 5 min before rPMS (baseline), just before the rPMS (Pre), and every 15 min for 75 min after the rPMS (Post 0, 15, 30, 45, 60, and 75). SICI and ICF were measured at Pre, Post 0, 15, 30, 45, 60, and 75 time points.

#### Experiment 4: Effects of rPMS on Motor Performance

The task has been previously used to assess ballistic motor performance and learning (Lundbye-Jensen et al., 2011; Yamaguchi et al., 2020). Participants performed a ballistic motor task involving the wrist extensor muscles on two separate days (at least 1 day apart) in a random order. To record the wrist extension torque, the strain gage (9E01-L31, San-ei, Tokyo, Japan) was placed on the head of the third metacarpal bone using a steel frame. Participants were instructed to perform an isometric maximal voluntary wrist extension against a strain gage with the highest possible acceleration in response to a visual GO cue, and subsequently return to the initial resting position within a total time window of 500 ms. A visual go cue, which consists of two vertical lines indicating a time window of 500 ms, was displayed in front of participants. The cursor moves from left to right in a horizontal line in the display, and the participants perform an isometric maximal voluntary wrist extension as quickly as possible when the cursor reaches the first vertical line. During these sessions assessing ballistic voluntary muscle contraction, the participants received no visual feedback regarding their motor performance. Participants performed two blocks of 10 trials with each trial lasting 3 s prior to 50 or 25 Hz rPMS, and three blocks (Post 0, 30, and 60) following rPMS. At the outset of the experiment and immediately following rPMS, the participants performed three test contractions to become accustomed to the task. A ballistic voluntary muscle contraction was quantified as the peak torque averaged over 10 trials, and expressed relative to the first baseline measure. Additionally, the root mean square value of rectified ECR EMGs for the first 100 ms of EMG onset (defined as increases of 100 μV above baseline EMG) was determined as a measure of voluntary muscle activation to complement the behavioral outcomes.

### Statistical Analysis

The Shapiro–Wilk test was used to determine whether the normalized MEP, SICI, ICF, Mmax, Hmax/Mmax, force, and EMG values were normally distributed. For Experiments 1 and 2, a repeated-measures mixed model ANOVA was used with conditions (50 Hz rPMS, 25 Hz rPMS and, 10 Hz rPMS), and time (T0, T5, T10, T15, and T20) representing two factors to compare the normalized MEP data for each frequency condition. When significant interaction or effects were determined, for *post hoc* comparisons, paired *t*-tests with Bonferroni adjustments were used for normally distributed data and Mann–Whitney U-test with Bonferroni adjustments were used for non-normally distributed data. To compare Mmax and Hmax/Mmax before (T0) and after rPMS (T20), a paired *t*-test for normally distributed data and Mann–Whitney U-test for non-normally distributed data were used. For Experiment 3, a repeated-measures mixed model ANOVA was used with condition (50 Hz rPMS and 25 Hz rPMS) and time (Pre, Post 0, Post 15, Post 30, Post 45, Post 60, and Post 75) representing two factors. When significant interaction or effects were determined, paired *t*-tests with Bonferroni adjustments were used for normally distributed data or Mann–Whitney U-test with Bonferroni adjustments for non-normally distributed data were used for *post hoc* comparisons. For Experiment 4, a repeated-measures mixed model ANOVA was used with conditions (50 Hz rPMS and 25 Hz rPMS) and time (Pre, Post 0, Post 30, and Post 60) representing two factors to compare wrist extension force and EMG activity. When significant interaction or effects were determined, for *post hoc* comparisons, paired *t*-tests with Bonferroni adjustments were used for normally distributed data and Mann–Whitney U-test with Bonferroni adjustments were used for non-normally distributed data. Statistical significance was set at *p* < 0.05 for all comparisons. All statistical analyses were performed using SPSS 26 (IBM, Armonk, NY, United States).

## Results

The Shapiro–Wilk test confirmed that all data except normalized MEP values were normally distributed. Therefore, normalized MEP values were analyzed using a non-parametric test.

### Experiment 1: Effects of rPMS Frequency on MEPs, M-Wave, and H-Reflex

#### MEPs

**FIGURE 2:**
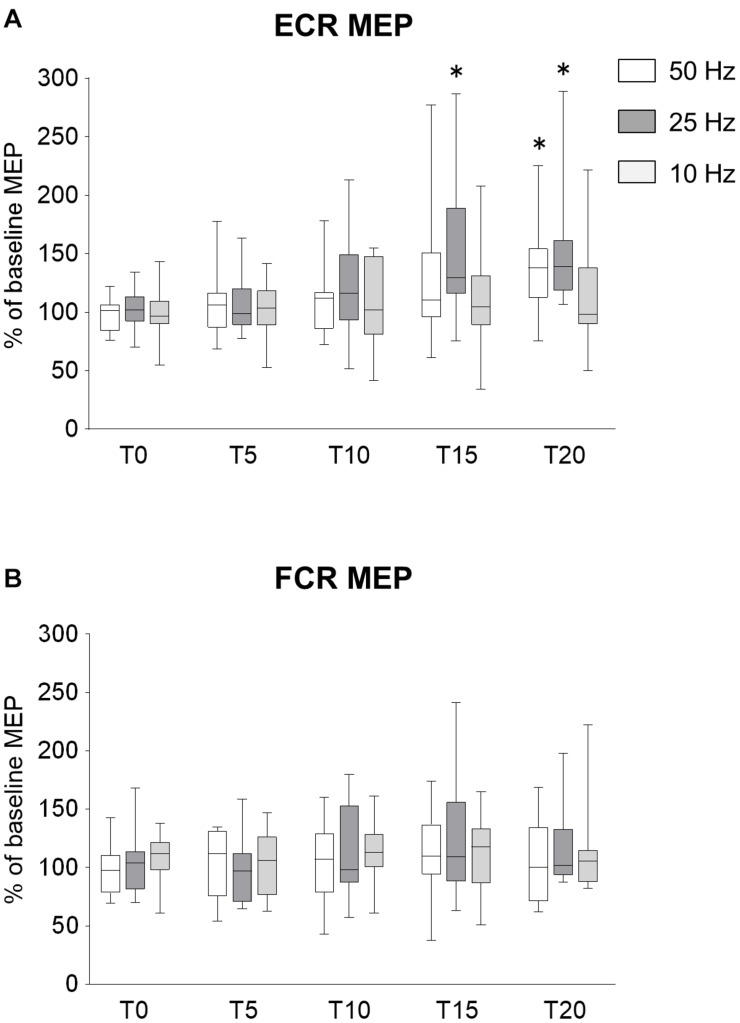
Effects of rPMS frequency on motor evoked potentials (MEPs). **(A)** MEP amplitudes of extensor carpi radialis (ECR) and **(B)** flexor carpi radialis (FCR) muscles were normalized to baseline amplitude. Each box plot indicates results following rPMS at 50 (white), 25 (dark gray), or 10 Hz (light gray). Median and interquartile ranges are represented by horizontal lines within boxes and whiskers (representing minimum and maximum values), respectively. Asterisks indicate significant differences compared to “T0” (*p* < 0.05).

To determine whether the frequency of rPMS have the different effects on corticospinal excitability and spinal network excitability, we applied rPMS using different frequencies. Time courses of normalized MEPs are shown in Figure 2. Figure 2A shows the changes in MEP of ECR, 50 Hz rPMS enhanced corticospinal excitability after a total of 20 min stimulation. 25 Hz rPMS also enhanced excitability after 15 min stimulation. No change was observed following 10 Hz rPMS after 20 min stimulation. Corticospinal excitability of the FCR was not changed in any of these conditions (Figure 2B).

There was no significant interaction between condition and time for the ECR (ANOVA, *F*_8,196_ = 1.071, *p* = 0.385). There was a significant main effect of condition (*F*_2,196_ = 6.515, *p* = 0.002) and time (*F*_4,196_ = 8.785, *p* < 0.001). For the FCR, there were no significant interactions (*F*_8,196_ = 0.377, *p* = 0.932) and no effects of rPMS (condition, *F*_2,196_ = 0.680, *p* = 0.508; time, *F*_4,196_ = 0.377, *p* = 0.932).

Comparing the MEPs of ECR among different conditions, there was a significant difference between 25 and 10 Hz rPMS (*p* = 0.001), but no significant difference was observed in other combinations. Comparing the time factor for each condition, 50 Hz rPMS significantly increased the normalized MEPs of the ECR at T20 compared to T0 (*p* = 0.005) and 25 Hz rPMS increased at T15 (*p* = 0.013) and T20 compared to T0 (*p* < 0.001), whereas no significant change was observed for 10 Hz rPMS (*p* > 0.05).

#### M-Waves and H-Reflex

The mean values of Mmax at T0 and T20 were 5.5 (SD 2.6) mV and 5.6 (SD 2.6) mV for 50 Hz rPMS, 6.7 (SD 1.7) mV and 6.9 (SD 1.6) mV for 25 Hz rPMS, and 7.8 (SD 3.0) mV and 8.0 (SD 3.0) mV for 10 Hz rPMS, respectively. Hmax/Mmax ratio was recorded in 6 of the 15 subjects, and the values of Hmax/Mmax at T0 and T20 were 0.14 (SD 0.03) and 0.12 (SD 0.02) for 50 Hz rPMS, 0.20 (SD 0.03) and 0.14 (SD 0.02) for 25 Hz rPMS, and 0.16 (SD 0.03) and 0.12 (SD 0.02) for 10 Hz rPMS, respectively. Paired *t*-tests revealed that there were no significant differences between T0 and T20 in Mmax amplitudes (*p* = 0.439 in 50 Hz; *p* = 0.618 for 25 Hz; *p* = 0.271 for 10 Hz) or Hmax/Mmax ratio (*p* = 0.369 for 50 Hz; *p* = 0.209 for 25 Hz; *p* = 0.145 for 10 Hz).

### Experiment 2: Effects of the Stimulus Number of rPMS on MEPs

#### MEPs

**FIGURE 3:**
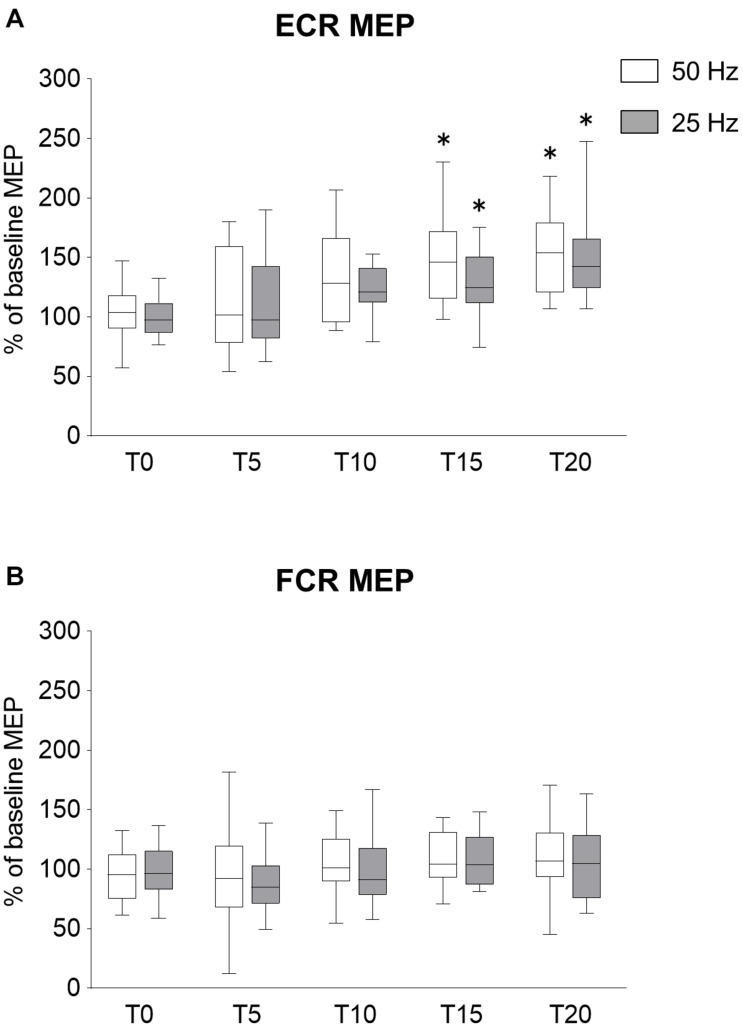
Effects of rPMS stimulus dose on motor evoked potentials (MEPs). **(A)** MEP amplitudes of extensor carpi radialis (ECR) and **(B)** flexor carpi radialis (FCR) were normalized to baseline amplitude. Each box plot indicates results following rPMS at 50 (white) and 25 Hz (dark gray). Median and interquartile ranges are represented by horizontal lines within boxes and whiskers (representing minimum and maximum values), respectively. Asterisks indicate significant differences compared to “T0” (*p* < 0.05).

In Experiment 1, we confirmed the effects of the rPMS frequency on corticospinal and spinal excitability, but the effect of increasing the number of stimuli was unclear. To examine the effects of increasing the number of stimuli on the changes in corticospinal excitability, the rPMS at 50 and 25 Hz was performed by increasing the number of stimuli compared to Experiment 1. The time courses of normalized MEPs are shown in Figure 3. No difference was found between 50 and 25 Hz rPMS. Figure 3A shows the changes in MEP of ECR, both 50 and 25 Hz rPMS enhanced corticospinal excitability after 15 min stimulation. Corticospinal excitability of the FCR was not altered in either condition (Figure 3B). There was no significant interaction between the condition and time for ECR (ANOVA, *F*_4,117_ = 0.450, *p* = 0.772). There was a significant main effect of time (*F*_4,117_ = 13.256, *p* < 0.001) but not of condition (*F*_1,117_ = 2.606, *p* = 0.109). Regarding the FCR, there were no significant interactions (*F*_4,117_ = 0.161, *p* = 0.958) or effects of rPMS (condition, *F*_1,117_ = 0.519, *p* = 0.473; time, *F*_4,117_ = 2.320, *p* = 0.061).

Comparing the time for each condition, 50 Hz rPMS significantly increased the normalized MEPs of the ECR at T15 (*p* = 0.005) and T20 compared to T0 (*p* < 0.001). Following 25 Hz rPMS, the normalized MEPs of ECR increased at T15 (*p* = 0.042) and T20 compared to T0 (*p* < 0.001).

#### M-Waves

The Mmax values at T0 and T20 were 5.5 (SD 2.6) mV and 5.6 (SD 2.6) mV for 50 Hz rPMS and 5.6 (SD 1.5) mV and 5.5 (SD 1.4) mV for 25 Hz rPMS, respectively. There were no significant differences between T0 and T20 in either condition (*p* = 0.369 for 50 Hz; *p* = 0.554 for 25 Hz).

### Experiment 3: Lasting Effects of rPMS on MEPs, SICI, and ICF

#### MEPs

**FIGURE 4:**
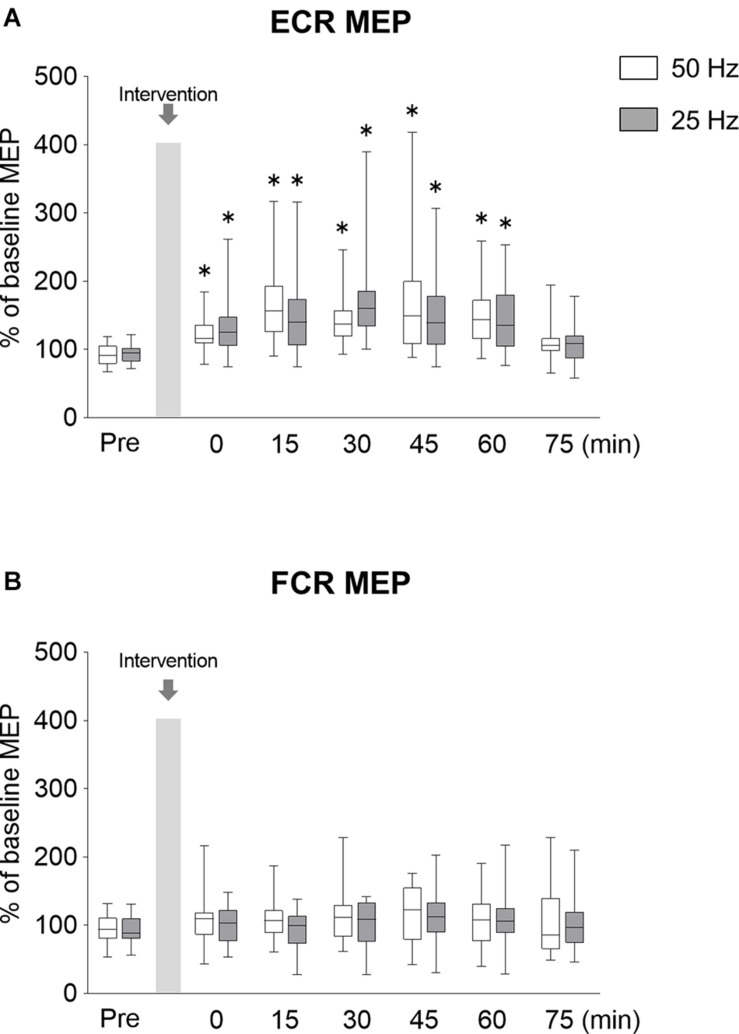
Lasting effects of rPMS on motor evoked potentials (MEPs). **(A)** MEP amplitudes of extensor carpi radialis (ECR) and **(B)** flexor carpi radialis (FCR) were normalized to baseline amplitude. Each box plot indicates that results following rPMS at 5 (white) and 25 Hz (dark gray). Median and interquartile ranges are represented by horizontal lines within boxes and whiskers (representing minimum and maximum values), respectively. Asterisks indicate significant differences compared to “Pre” (*p* < 0.05).

In order to investigate the lasting effects of rPMS on MEPs, SICI, and ICF, we examined these parameters over a time course after rPMS was performed. The time courses of normalized MEP are shown in Figure 4. Figure 4A shows the changes in MEP of ECR, both 50 and 20 Hz rPMS enhanced corticospinal excitability immediately after rPMS, and lasting effects were observed up to 60 min following the rPMS. Corticospinal excitability of the FCR was not changed in either condition (Figure 4B). There was no significant interaction between condition and time for the ECR (ANOVA, *F*_6,247_ = 1.519, *p* = 0.172). There was a significant main effect of time (*F*_6,247_ = 14.408, *p* < 0.001), but not of condition (*F*_1,247_ = 0.001, *p* = 0.972). For the FCR, there were neither significant interactions (*F*_6,247_ = 0. 321, *p* = 0.926) nor effects of rPMS (condition, *F*_1,247_ = 2.051, *p* = 0.15; time, *F*_6,247_ = 1.477, *p* = 0.187).

Compared to the normalized MEP values at the Pre time point, the normalized MEP of ECR following 50 Hz rPMS was significantly increased at the Post 0 (*p* = 0.001), Post 15 (*p* < 0.001), Post 30 (*p* < 0.001), Post 45 (*p* < 0.001), and Post 60 (*p* < 0.001) time points, while no significant difference was observed at the Post 75 time point (*p* = 0.196). Following 25 Hz rPMS, the normalized MEP of ECR was significantly increased at the Post 0 (*p* = 0.001), Post 15 (*p* < 0.001), Post 30 (*p* < 0.001), Post 45 (*p* < 0.001) and Post 60 (*p* = 0.001) time points, with no significant difference at the Post 75 time point (*p* = 0.158).

#### SICI and ICF

**FIGURE 5:**
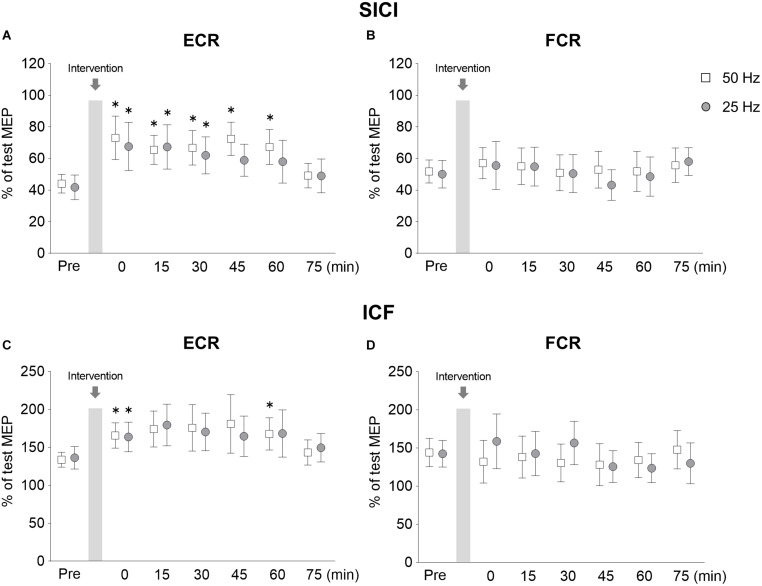
Lasting effects of rPMS on short-interval intracortical inhibition (SICI) and intracortical facilitation (ICF). Conditioned MEP amplitudes in the ECR **(A,C)** and FCR **(B,D)** were normalized to mean test MEP amplitudes to calculate SICI (inter-stimulus interval: 2.5 ms) **(A,B)** and ICF (inter-stimulus interval: 10 ms) **(C,D)**. The mean values and 95% confidence interval obtained from 20 participants are indicated. Each symbol indicates results following rPMS at 50 (white box) and 25 Hz (dark gray circle). Asterisks indicate significant differences compared to “Pre” (*p* < 0.05).

The time courses of SICI and ICF are shown in Figure 5. Figure 5A shows the changes in SICI of ECR, the SICI was significantly weaker immediately after 50 Hz rPMS, and lasting effects were observed for up to 60 min. The SICI was also weaker immediately after 25 Hz rPMS, and lasting effects were observed for up to 30 min. Figure 5B shows the changes in SICI of FCR, no apparent changes in SICI were observed following either 50 or 25 Hz rPMS. These results were supported by ANOVA, indicating significant main effects of condition (*F*_1,247_ = 4.789, *p* = 0.030) and time (*F*_6,247_ = 9.446, *p* < 0.001), but no significant interaction between condition and time for the ECR (*F*_6,247_ = 0.770, *p* = 0.594). In the FCR, there was neither significant interaction (*F*_6,247_ = 0. 369, *p* = 0.898) nor effects of rPMS (condition, *F*_1,247_ = 0.785, *p* = 0.376; time, *F*_6,247_ = 1.207, *p* = 0.303).

Following 50 Hz rPMS, the SICI at the Post 0 (*p* = 0.008), Post 15 (*p* = 0.014), Post 30 (*p* = 0.031), Post 45 (*p* = 0.001), and Post 60 (*p* = 0.001) time points was significantly weaker than that at the Pre time point. Compared to the SICI at the Pre time point, no significant differences were observed at the Post 75 time point (*p* > 0.05). Following 25 Hz rPMS, SICI at the Post 0 (*p* = 0.001), Post 15 (*p* = 0.003), and Post 30 (*p* = 0.026) time points were significantly weaker than at the Pre time point. Compared to the SICI at the Pre time point, no significant difference was observed after Post 45 (*p* > 0.05).

When the SICI values were compared between rPMS conditions at each time point, the 50 Hz rPMS was found to enhance the inhibition at the Post 45 time point (*p* = 0.046). No significant differences were observed between 50 and 25 Hz rPMS at the other time points examined (*p* > 0.05).

Figure 5C shows the changes in ICF of ECR, the ICF was stronger immediately after 50 Hz rPMS, and lasting effects were observed for up to 60 min. ICF was stronger immediately after 25 Hz rPMS as well, though the lasting effects were observed only up to 15 min. No apparent changes were observed in ICF of the FCR following either 50 or 25 Hz rPMS (Figure 5D).

ANOVA indicated that the differences in the ICF of the ECR were dependent on time (*F*_6,247_ = 4.296, *p* < 0.001), but not condition (*F*_1,247_ = 0.024, *p* = 0.877) and no significant interaction occurred between condition and time (*F*_6,247_ = 0.302, *p* = 0.936). In the FCR, there were neither significant interactions (*F*_6,247_ = 1.137, *p* = 0.341) nor significant effects (condition, *F*_1,247_ = 0.337, *p* = 0.562; time, *F*_6,247_ = 0.840, *p* = 0.540).

Following 50 Hz rPMS, the ICF of the ECR at the Post 0 (*p* = 0.026) and Post 60 (*p* = 0.007) time points was significantly stronger than that at the Pre time point, but no significant difference was observed at the other time points (*p* > 0.05). Following 25 Hz rPMS, the ICF of the ECR at the Post 0 time point (*p* = 0.026) was significantly stronger than that at the Pre time point, but no significant difference was observed at the Post 15 time point (*p* > 0.05).

#### M-Waves

The Mmax values at Pre and Post 75 time points were 7.7 (SD 2.8) mV and 7.6 (SD 3.3) mV for 50 Hz and 7.4 (SD 2.8) mV and 7.6 (SD 2.7) mV for 25 Hz, respectively. There was no significant difference between the Pre and Post values in each condition (*p* = 0.917 for 50 Hz; *p* = 0.863 for 25 Hz).

### Experiment 4: Effects of rPMS on Motor Performance

**FIGURE 6:**
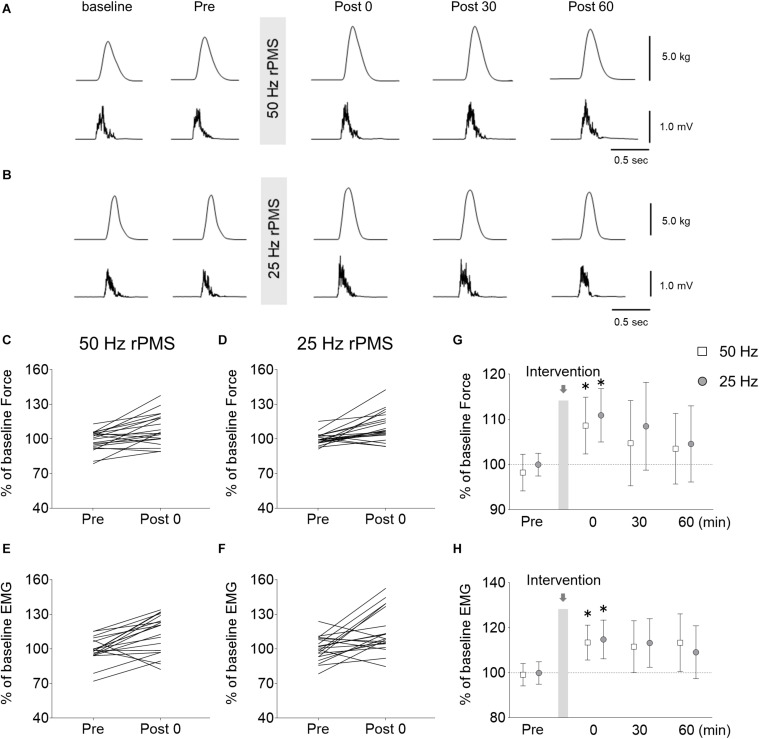
Changes in force and electromyogram (EMG) during wrist ballistic movements. Raw traces of force (upper) and rectified EMG from the ECR (lower) obtained from a subject during brief, fast, isometric voluntary wrist extension movements before and after rPMS at 50 **(A)** and 25 Hz **(B)**. Each waveform represents the average of 10 trials. Individual changes in force **(C,D)** and EMGs **(E,F)** during the movements before and immediately after rPMS are shown. Group data of force **(G)** and EMGs **(H)** are also shown. The mean values and 95% confidence intervals obtained from 20 participants are indicated. Asterisks indicate significant differences compared to “Pre” (*p* < 0.05).

To investigate whether rPMS could affect motor performance, we measured force and EMG activity using a ballistic motor task involving the wrist extensor muscles. Force and EMG activity from the ECR are shown in Figure 6. No difference was found between 50 and 25 Hz rPMS. The normalized peak force and EMG activity values increased immediately after 50 Hz rPMS (Figures 6G,H). Following 25 Hz rPMS, the normalized peak force and EMG activity values were also increased. There was no significant interaction between condition and time for the force (ANOVA, *F*_3,133_ = 0.076, *p* = 0.973). There was a significant main effect of time (*F*_3,133_ = 4.766, *p* = 0.003) but not of condition (*F*_1,133_ = 1.148, *p* = 0.286). For the EMG, there was no significant interaction between condition and time (ANOVA, *F*_3,133_ = 0.232, *p* = 0.874). There was a significant main effect of time (*F*_3,133_ = 5.280, *p* = 0.002) but not of condition (*F*_1,133_ = 0.002, *p* = 0.969).

Following 50 Hz rPMS, the normalized peak force (*p* = 0.004) and EMG values (*p* = 0.003) were stronger immediately after rPMS than at the Pre time point, but no significant change could be detected at subsequent time points. Following 25 Hz rPMS, the normalized peak force (*p* = 0.005) and EMG values (*p* = 0.021) were stronger immediately after rPMS than at the Pre time point, but similarly, no significant change could be detected at subsequent time points.

## Discussion

The present study demonstrated that the rPMS over the wrist extensor muscle at 50 and 25 Hz, but not 10 Hz, increases the cortical excitability of the targeted area alone, without changing the excitability in the spinal circuit. In both frequency conditions, although the number of stimuli was different, the increase in cortical excitability was similarly induced following more than 15 min of rPMS, and the effects lasted up to 60 min after rPMS. In addition, the attenuation of intracortical inhibition and enhancement of ICF were observed following rPMS. In the behavioral experiment, rPMS induced an increase in ballistic wrist extension force and EMG activity of the ECR muscle. These results suggest that 15 min of rPMS with 25 Hz or more may be an effective way to promote rehabilitation training by enhancing cortical excitability and motor outputs, particularly in stroke patients or patients with neurodegenerative disorders.

### The Dose- or Frequency-Dependent Effects of rPMS on Cortical Excitability Changes

Previous studies were unclear on what might affect rPMS-induced cortical excitability changes, either dose or frequency of stimulation. The results of Experiment 1, which aligned the total number of stimuli, displayed a significant increase in MEPs following both 50 and 25 Hz rPMS. At 50 Hz rPMS, a significant increase in MEP was observed after 8,000 stimuli over 20 min, similar to that observed after 6,000 stimuli over 15 min at 25 Hz rPMS. However, no increase in MEP was observed following 10 Hz rPMS, even up to 8,000 stimuli over 20 min. Therefore, in order to induce these plastic changes in the MEP, the frequency of rPMS had to be at least 25 Hz or more, and the number of stimuli required was greater than 6,000. Per the results of Experiment 2, when 7,500 stimuli were applied over 5 min/session at 50 Hz rPMS, an increase in MEP was observed after 15 min. Since the same result was obtained by 25 Hz rPMS, although the number of stimuli was less than in the 50 Hz rPMS, the intervention time required to induce these plastic changes was at least 15 min. Therefore, it was found that plastic changes in cortical excitability are induced in a frequency-dependent manner when the intervention timeframe is fixed. This is supported by an animal study indicating that the strength of synaptic connections change in a stimulus frequency-dependent manner (Dudek and Bear, 1992). These authors demonstrated that 50 Hz stimulation induces long-term synaptic potentiation in rat hippocampal neurons, but not 10 Hz stimulation, when the number of stimuli is fixed. It is therefore speculated that frequency-dependent changes shown in the present study also occur in the motor cortex (Iriki et al., 1989). In addition, per Experiment 1, the excitability of the spinal circuit did not change significantly following rPMS, suggesting that plastic changes induced by rPMS are caused by modulation of the transmission efficiency in the motor cortex rather than in the spinal cord.

### The Mechanism of rPMS-Induced Changes in Cortical Excitability

A previous study indicated that the discharge rates of group Ia afferents from the wrist extensor muscles are 20–50 Hz during voluntary wrist movement (Kakuda and Nagaoka, 1998). Thus, afferent inputs at 50 and 25 Hz rPMS, which are similar to afferent discharges from muscle spindles, might increase signaling in the sensorimotor cortex. Such a physiological-like sensory input may effectively enhance cortical excitability. However, this does not mean that 10 Hz rPMS will never induce an increase in cortical excitability; previous studies have shown that peripheral nerve stimulation at 10 Hz induced an increase in MEP, but required an intervention time of over 2 h (Ridding et al., 2000; Kaelin-Lang et al., 2002). McKay et al. (2002) reported that an intervention time of at least 45 min was required to induce these changes. Therefore, the reason that the increase in amplitude of MEPs could not be induced in our present study may be due to insufficient dose or intervention time. It is possible that an increase in MEP can be induced by increasing the dose or intervention time even with 10 Hz rPMS.

### The Lasting Effects of rPMS on Cortical Excitability

Based on the results of Experiments 1 and 2, we evaluated lasting effects following 50 and 25 Hz rPMS for 15 min. An increase in MEP was observed immediately after rPMS, and lasting effects were observed for up to 60 min following both 50 and 25 Hz rPMS. Moreover, in agreement with a previous study (Gallasch et al., 2015), we found that SICI was weaker and ICF was stronger following rPMS. SICI reflects inhibitory interneuron excitability via GABA_A_ receptors (Ziemann et al., 2015); although the mechanism of action of ICF is unclear, it likely displays the excitability of facilitatory interneurons via the glutamate circuit (Ziemann et al., 2015). Gallasch et al. (2015) reported that the attenuation of SICI and enhancement of ICF can last up to 30 min after 20 min of 25 Hz rPMS. In the present study, with an intervention duration of 15 min, the 25 Hz rPMS showed lasting effects up to 15 min. This difference is likely due to the duration of the intervention. In addition, the present study showed that after 50 Hz rPMS, although the duration of the intervention was 15 min, lasting effects were observed for up to 60 min. Thus, the duration of the intervention and the stimulus frequency may affect these lasting effects.

### Stimulus Intensity of rPMS

In the present study, the stimulus intensity of rPMS was set at 120% of the MT. This relatively weak stimulation activates low-threshold fibers, e.g., group Ia and Ib afferents and alpha motor axons. Since rPMS is thought to stimulate tissue deeper than electrical stimulation (Polson et al., 1982), it is assumed that the cutaneous nerves at the site of stimulation site are barely activated. Therefore, the group I afferents, which are ascending inputs, should be responsible for the increase in cortical excitability. Interestingly, previous studies have reported that MEP amplitude was unchanged (Ridding et al., 2000) or decreased following peripheral nerve electrical stimulation below MT intensity (Mima et al., 2004; Tinazzi et al., 2005; Schabrun et al., 2012; Sasaki et al., 2017). Sasaki et al. (2017) demonstrated that electrical stimulation to the median nerve at the wrist above the MT intensity increases MEP amplitudes, whereas that with the intensity below MT decreases MEPs. They also demonstrated that cutaneous nerve stimulation to the index finger, derived from the median nerve, decreases MEPs. Thus, rPMS may be more effective in increasing the cortical excitability via increased ascending input than electrical stimulation.

### The Behavioral Effects of rPMS

An increase in wrist extension force and EMG activity during ballistic wrist extension movements was observed following 50 and 25 Hz rPMS in Experiment 4. The simplest explanation for this is that these increases might be caused by an increase in cortical excitability-evoked temporally synchronized descending volleys, and a greater number of spinal motoneurons are excited synchronously. This result is consistent with previous reports (Ridding et al., 2000; Conforto et al., 2002, 2018; Kaelin-Lang et al., 2002). Conforto et al. (2002, 2018) showed that the repetitive electrical stimulation to peripheral nerves, which induced an increase in cortical excitability in healthy individuals (Ridding et al., 2000; Kaelin-Lang et al., 2002), improved not only pinch strength but also hand motor function in stroke patients with movement disorders. Stroke patients had a decreased number of functioning motor units (McComas et al., 1973) and decreased firing frequency of those motor units (Rosenfalck and Andreassen, 1980; Tang and Rymer, 1981); therefore, it is likely that rPMS could be more effective in stroke patients exhibiting decreased motor cortex excitability.

### Limitations

The sample size of the current study was relatively small, although similar to previous study applying rPMS over wrist muscles (Gallasch et al., 2015). In the future, investigation needs to be carried out based on power analysis. In addition, this study was conducted in healthy participants. It is necessary to investigate the effects of rPMS on cortical excitability and motor performance in patients with CNS lesions. Furthermore, we could record the H-reflex at rest in only 6 of 15 participants in Experiment 1. Although no participants showed a change in amplitude largely before and after the rPMS, the sample size may be small to conclude that there is no change in the excitability of spinal networks. Another limitation is that the stimulus intensity was set at 120% of the MT; there may be more effective stimulus intensities to increase cortical excitability, since the effect on cortical excitability changes with intensity (Sasaki et al., 2017). Further experiments are required to investigate the effects of various stimulus intensities of rPMS, and to compare effects between magnetic and electrical stimulation intervention on cortical excitability.

## Conclusion

The present study demonstrated that the rPMS to ECR muscle enhances cortical excitability of the relevant area rather than altering the excitability in spinal networks, and has the potential to improve wrist motor output. Thus, 15 min of rPMS of ≥25 Hz would be a useful technique in the rehabilitation of motor function following lesions of the CNS by enhancing the cortical excitability of residual motor circuits.

## Data Availability Statement

The raw data supporting the conclusions of this article will be made available by the authors, without undue reservation.

## Ethics Statement

The studies involving human participants were reviewed and approved by the Ethics Committee of Yamagata Prefectural University Health of Sciences. The patients/participants provided their written informed consent to participate in this study.

## Author Contributions

MN and TY conceived and designed the experiments, and drafted the manuscript. MN, NK, and KY recruited participants and collected the data. MN analyzed the data. MN, NK, TK, DK, SN, and TY interpreted the results of experiments. ST constructed the program for data collection. All authors approved the final version of the submitted manuscript.

## Conflict of Interest

The authors declare that the research was conducted in the absence of any commercial or financial relationships that could be construed as a potential conflict of interest.

## Abbreviations

aMT: active motor threshold
CNS: central nervous system
EMG: electromyogram
ECR: extensor carpi radialis
FCR: flexor carpi radialis
H-reflex: Hoffmann-reflex
ICF: intracortical facilitation
MEP: motor evoked potential
M-wave: direct motor response
rMT: resting motor threshold
rPMS: repetitive peripheral magnetic stimulation
SD: standard deviation
SICI: short-interval intracortical inhibition
TMS: transcranial magnetic stimulation.
